# Deep learning for risk stratification of thymoma pathological subtypes based on preoperative CT images

**DOI:** 10.1186/s12885-024-12394-4

**Published:** 2024-05-28

**Authors:** Wei Liu, Wei Wang, Ruihua Guo, Hanyi Zhang, Miaoran Guo

**Affiliations:** 1 School of Health Management, China Medical University, Shenyang, Liaoning China; 2 Department of Radiology, Shengjing Hospital of China Medical University, Shenyang, Liaoning China; 3 School of Computer Science, The University of Sydney, Sydney, Australia; 4 Department of Radiology, Liaoning Cancer Hospital & Institute, Shenyang, Liaoning China; 5 Department of Radiology, The First Hospital of China Medical University, Shenyang, Liaoning China

**Keywords:** Thymoma, Tumor segmentation, Risk stratification, Deep learning, Clinical decision support system

## Abstract

**Objectives:**

This study aims to develop an innovative, deep model for thymoma risk stratification using preoperative CT images. Current algorithms predominantly focus on radiomic features or 2D deep features and require manual tumor segmentation by radiologists, limiting their practical applicability.

**Methods:**

The deep model was trained and tested on a dataset comprising CT images from 147 patients (82 female; mean age, 54 years ± 10) who underwent surgical resection and received subsequent pathological confirmation. The eligible participants were divided into a training cohort (117 patients) and a testing cohort (30 patients) based on the CT scan time. The model consists of two stages: 3D tumor segmentation and risk stratification. The radiomic model and deep model (2D) were constructed for comparative analysis. Model performance was evaluated through dice coefficient, area under the curve (AUC), and accuracy.

**Results:**

In both the training and testing cohorts, the deep model demonstrated better performance in differentiating thymoma risk, boasting AUCs of 0.998 and 0.893 respectively. This was compared to the radiomic model (AUCs of 0.773 and 0.769) and deep model (2D) (AUCs of 0.981 and 0.760). Notably, the deep model was capable of simultaneously identifying lesions, segmenting the region of interest (ROI), and differentiating the risk of thymoma on arterial phase CT images. Its diagnostic prowess outperformed that of the baseline model.

**Conclusions:**

The deep model has the potential to serve as an innovative decision-making tool, assisting on clinical prognosis evaluation and the discernment of suitable treatments for different thymoma pathological subtypes.

**Key Points:**

• This study incorporated both tumor segmentation and risk stratification.

• The deep model, using clinical and 3D deep features, effectively predicted thymoma risk.

• The deep model improved AUCs by 16.1pt and 17.5pt compared to radiomic model and deep model (2D) respectively.

**Supplementary Information:**

The online version contains supplementary material available at 10.1186/s12885-024-12394-4.

## Introduction

 Thymoma is the most common anterior mediastinal tumor, originating from the thymic epithelium. Its clinical manifestations are diverse, and can present as chest pain, coughing, hemoptysis, dyspnea, dysphagia, and other symptoms. According to the National Cancer Institute in the United States, the incidence rate of thymoma in the US is approximately 1.3 per 100,000 population, with around 400 new cases each year [1]. While thymoma is relatively uncommon, it remains the most prevailing type of mediastinal tumor. Pathological classification provides important information about the biological behavior of thymomas, consequently informing both clinical prognosis evaluation and the selection of appropriate treatments. The accurate classification of thymoma subtypes within imaging data is essential due to its pivotal role in treatment planning and prognostic anticipation. According to the World Health Organization (WHO) standards, thymomas are classified into pathological subtypes, namely A, AB, B1, B2, and B3 [2]. Generally, types A, AB, and B1 correspond to a lower risk profile and a favorable prognosis, whereas types B2 and B3 are associated with a higher risk and a less favorable prognosis [3].

Machine learning has recently displayed significant potential in diverse medical analytical tasks. These encompass tumor classification [4], prognosis prediction [5], detection of nodules [6], determination of gene mutation status [7], and lesion segmentation [8], among others [9–12]. However, there has been relatively limited progress in the evaluation of thymoma risk using these techniques. Previous studies have employed machine learning models to classify thymomas as high or low risk based on either unimodal or multi-modal CT imaging and clinical data [13–15]. For instance, Shang et al. [16] conducted a study to assess the diagnostic accuracy of machine learning models using multiple classifiers. Their study focused on leveraging non-enhanced CT radiomics features to differentiate between anterior mediastinal cysts and thymomas, as well as between high-risk and low-risk thymomas. More sophisticated methodologies encompass the incorporation of deep learning features extracted from a convolutional neural network (CNN). Nakajo et al. [17] constructed a prediction model by combining 107 radiomic features and 1024 deep learning features, employing six distinct machine learning algorithms to forecast pathological risk subtypes. Among these algorithms, the logistic regression model displayed the highest AUC and accuracy, with values of 0.900 and 0.810, respectively, for the prediction of thymic carcinoma.

However, previous studies on risk prediction of thymoma subtypes required the manual segmentation of tumor, involving radiologists delineating the regions of interest. Furthermore, these studies primarily emphasized on radiomic features or 2D deep features, rather than 3D deep features extracted from CT images. To overcome these limitations, this study proposed a deep learning framework for tumor risk stratification based on 3D deep features. We have integrated tumor segmentation and subtype high risk and low risk prediction into a framework, enhancing its practical utility.

## Materials and methods

### Patient selection

The retrospective study was approved by the institutional review board of Shengjing hospital, which waived the requirement for informed patient consent. The analysis was conducted on thymoma patients who had undergone surgical resection at our hospital. These patients received pathological confirmation and provided the necessary clinical data from January 2015 to October 2019. The documented clinical characteristics encompassed gender, age, and symptoms such as chest discomfort, chest pain, cough, and the presence of myasthenia gravis.

### Inclusion and Exclusion criteria

**Fig. 1:**
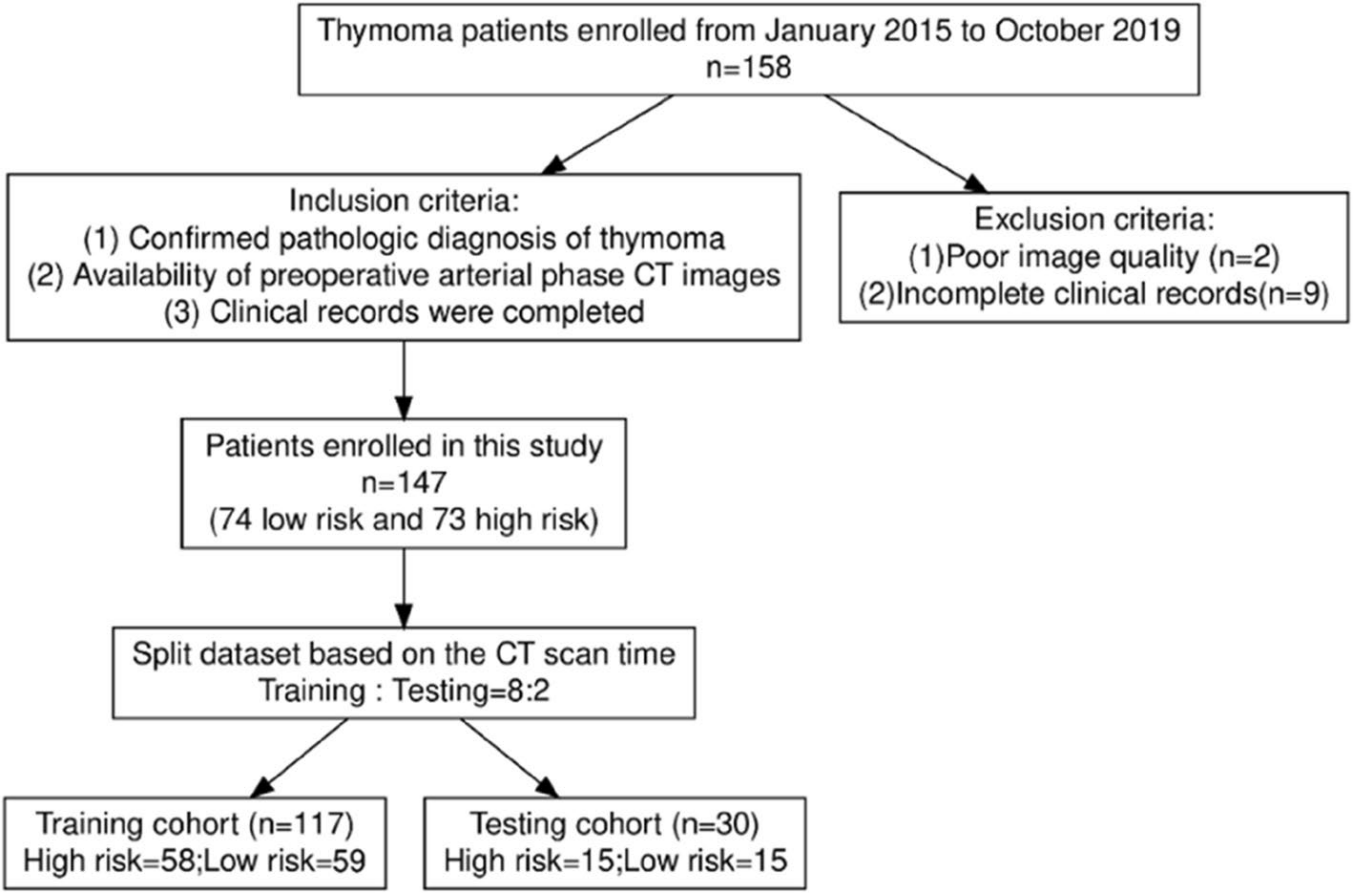
Flow diagram of cohort selection. After applying the inclusion and exclusion criteria, we ultimately obtained 147 samples, including 73 high-risk samples and 74 low-risk samples. The number of cases in the high-risk and low-risk groups was sampled to be evenly distributed by chance. The dataset was stratified at a ratio of 8:2, with 117 samples in the training set. As 117 is an odd number, the training set included 58 high-risk and 59 low-risk samples. The test set consisted of 30 samples, with an equal number of high-risk and low-risk samples, each being 15

The study cohort was selected based on the specified criteria as illustrated in Fig. 1.

#### Inclusion criteria

- Confirmed pathologic diagnosis of thymoma.
- Availability of preoperative arterial phase CT images.
- Clinical records were completed.

#### Exclusion criteria

- Poor image quality.
- Incomplete clinical records.

**Fig. 2:**
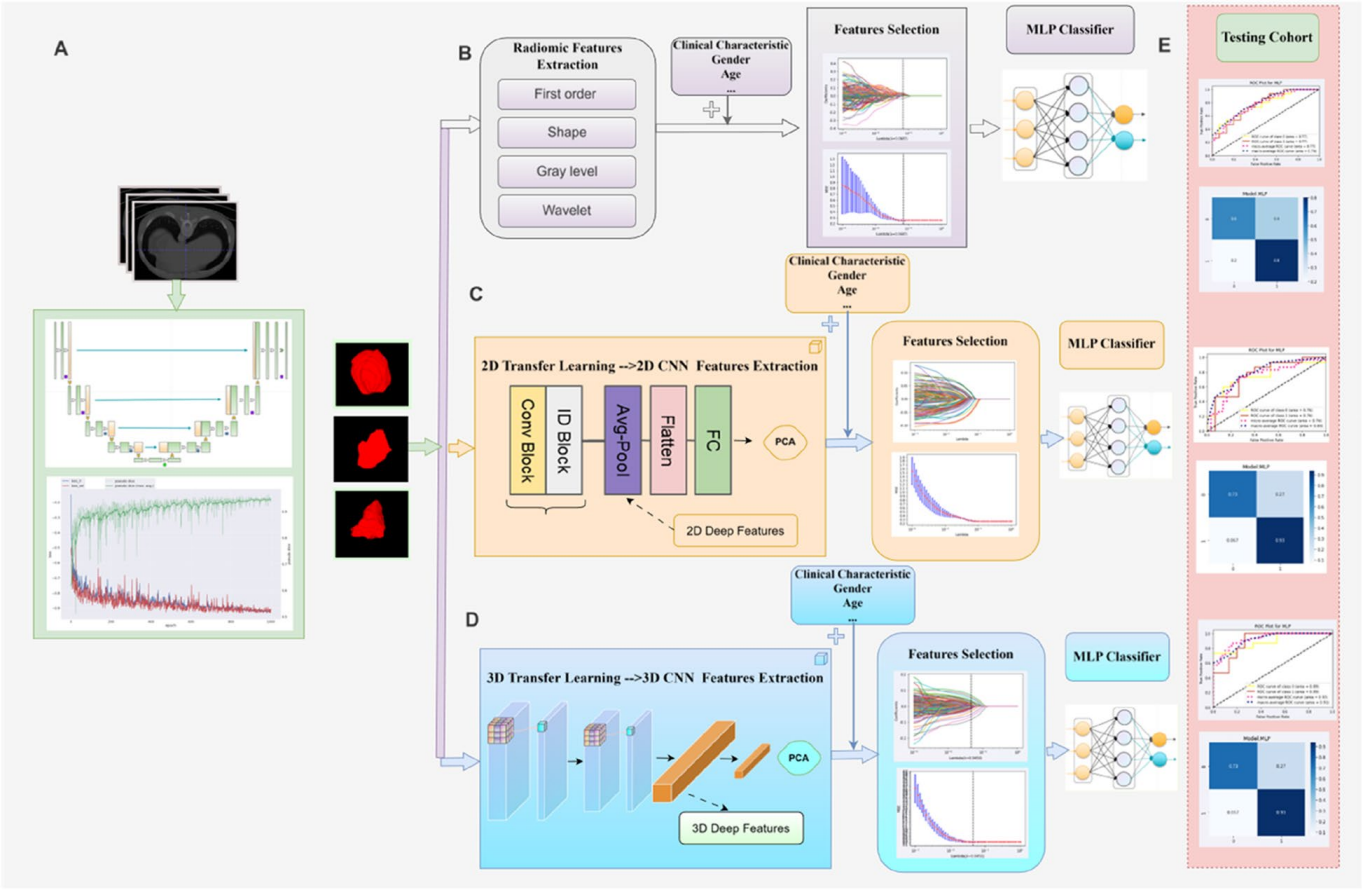
The overall pipeline of this study. **A** The framework takes an arterial CT scan as input and includes a three-dimensional (3D) tumor area segmentation network with nnU-Net architecture. We obtained voxel-level segmentation labels and cropped the three-dimensional ROI of the tumor. **B** We extracted the radiomic features of the ROI. The LASSO method was used to select the features. Following feature selection, we constructed an MLP Classifier based on the radiomic features and clinical characteristics. **C** We utilized deep transfer learning technology based on the 2D ResNet50 network. LASSO was applied in sequence for dimensionality reduction and feature selection. Finally, an MLP Classifier was constructed based on the 2D deep features and clinical characteristics. **D** We utilized deep transfer learning technology based on the 3D ResNet50 network. The pre-trained weights were obtained from 3D MedicalNet, a medical network, and used to extract the 3D deep-learning features of the ROI. Subsequently, LASSO was applied in sequence for dimensionality reduction and feature selection. Finally, an MLP Classifier was constructed based on the 3D deep features and clinical characteristics. **E** In the testing cohort, the predicted probabilities of high and low risk of tumor were the outputs. We performed result analysis using ROC curves, the AUC, and decision curve analysis

The overall pipeline of this study was shown in Fig. 2:

The pipeline incorporated a tumor segmentation network and a risk stratification network (high risk and low risk). The tumor segmentation network used an arterial CT scan as input to obtain voxel-level segmentation labels and crop the three-dimensional the region of interest (ROI) of the tumor. In the risk stratification network, the deep model utilized transfer learning technology to extract the 3D deep-learning features of the ROI, leveraging the pre-trained weights of the ResNet50 3D model. The extracted 2048 3D deep features were dimensionally reduced to 128 using PCA, to enhance training efficiency and optimize performance. The deep features were then concatenated with the clinical characteristics, and LASSO was utilized for feature selection finally. Ultimately, an MLP classifier was built based on the 3D deep features and clinical characteristics. For comparison, we also extracted radiomic features from the ROI and 2D deep features from the maximum cross-section of the ROI. We then constructed two MLP classifiers using these radiomic features and clinical characteristics, and 2D deep features and clinical characteristics respectively. In the testing cohort, the performance of the models was evaluated using receiver operating characteristic (ROC) curves, the area under the curve (AUC), and decision curve analysis(DCA).

### Image acquisition and lesion segmentation

The imaging was conducted using a 320-channel scanner (Aquilion ONE 640; Canon Medical Systems) and a 256-channel scanner (Brilliance 128; Philips Medical Systems). The imaging parameters employed were as follows: tube current ranged from 80 to 230 mA, tube voltage was set at 120 kV, slice thickness was between 1 and 3 mm, field of view (FOV) was 500 mm, and detector pitch spanned 0.75–1.172 mm. Each patient received 80–100 mL of a non-ionic iodinated contrast agent (300 mgI/mL) at a flow rate of 2.5-3.0 mL/s. The average imaging delays stood at 30–40 s for the arterial phase and 65–70 s for the portal venous phase.

To facilitate the training of the segmentation model, a total of 117 arterial phase CT images were manually annotated and employed as the training dataset. To ensure the accuracy and reliability of the manual annotations, we implemented a multi-check method. Each image was independently annotated by two doctors, who would then compare and discuss their annotation results to resolve any discrepancies. Additionally, we employed the Intraclass Correlation Coefficient (ICC) for evaluation (> 0.85). Notably, the annotators remained blinded to the histopathology results during this procedure. The ROI relating to the tumor were manually delineated on each image slice using 3D Slicer (version 4.11) [18].

### Model construction and validation

We constructed a two-stage framework that includes tumor segmentation network and risk stratification network.

#### Tumor segmentation

The nnU-Net [19] network was employed as the segmentation network in this study. The nnU-Net was a self-adapting framework designed for semantic segmentation of medical imaging data. It was capable of producing reliable and high-quality results without requiring manual network design or task-specific tuning. 117 annotated arterial phase CT images were used as the input data. A 5-fold cross-validation method was employed to train the segmentation network. The dice coefficient and loss curves were applied to evaluate segmentation performance [20, 21].

#### Risk stratification network

We utilized deep transfer learning technology based on the 3D ResNet50 [22] network to extract the 3D deep features of the ROI from the result of segmentation network. The pre-trained weights were from MedicalNet [23], which provided an effective way to train deep learning models for medical image analysis by leveraging the power of pre-training and transfer learning. The “avgpool” layer’s features from the 3D resnet50 convolutional network were used as the deep features. A total of 2048 3D deep features from arterial phase CT images were extracted and dimensionally reduced to 128 using PCA. The deep features were then concatenated with the clinical characteristics. To enhance the generalization ability of the model, LASSO was used for feature selection. Deep model was carried out using the MLP classifier [24], 128 deep features and 7 clinical features as an input. After feature selection by LASSO, Ultimately, 35 deep features and 1 clinical feature (gender) were used to construct the prediction model. Additionally, A radiomics model and deep model (2D) were built for comparison [25]. 864 radiomic features and 7 clinical features as an input, 7 radiomics and 1 clinical feature were selected by LASSO and used to construct a radiomic model with MLP classifier. 2048 2D deep features and 7 clinical features as an input, 35 2D deep and 1 clinical feature were selected by LASSO and used to construct a deep model (2D) with MLP classifier. AUC, Accuracy, sensitivity and specificity were used to evaluate the performance of the models. The network architecture was implemented by python software and pytorch library on a server with two GPUs (NVIDIA RTX 3090).

### Statistical analysis and performance evaluation

In this study, the dataset was divided by temporal validation. It was a form of external validation, which involves the independent testing of a model’s performance on subsequent patients at the same center. This method was considered to be a stronger design compared to randomly splitting a single dataset to assess model performance, as it allows for the consideration of nonrandom variations between datasets [26, 27]. Divided the training cohort (January 30, 2015 to November 23, 2018) and testing cohort (November 25, 2018 to October 12, 2019) based on the CT scan time. The training cohort comprised 117 patients (80%), while the testing cohort had 30 patients (20%) [28, 29].

Statistical analyses were performed using R software, version 4.2.2, along with MSTATA software (www.mstata.com). Categorical variables were compared using the chi-square test. The dice was calculated to evaluate the performance of segmentation network. The AUC, confusion matrix, calibration curve and decision curve were applied to evaluate prediction model performance.

## Results

### Descriptive statistics for baseline characteristics

The baseline information was summarized in Table 1. The gender distribution revealed that among the total sample size (*N* = 147), 53% were male (39 out of 73) and 35% were male (26 out of 74), showing a statistically significant difference (*p*-value: 0.026). No significant difference was observed for age between the two groups, as indicated by a *p*-value of 0.887. In terms of cough symptoms, both groups had a similar distribution, with 82% (60 out of 73) and 81% (60 out of 74) reporting the absence of cough. The presence of myasthenia gravis was also evenly distributed, with 21% (15 out of 73) in both groups. However, there was a notable difference in the presence of chest pain, with 32% (23 out of 73) of the first group and 18% (13 out of 74) of the second group reporting this symptom, resulting in a significant *p*-value of 0.049. Chest distress and other symptoms did not show any statistically significant difference between the two groups.

**Table 1:** Patient demographics and baseline characteristics

Characteristic	label		*p*-value^2^
	High risk, *N* = 73^1^	Low risk, *N* = 74^1^	
**Gender**			0.026
Male	39 (53%)	26 (35%)	
Female	34 (47%)	48 (65%)	
**Age**			0.887
-45	15 (21%)	13 (18%)	
46–61	37 (51%)	38 (51%)	
62-	21 (29%)	23 (31%)	
**Cough**			0.862
No	60 (82%)	60 (81%)	
Yes	13 (18%)	14 (19%)	
**Myasthenia gravis**			0.967
No	58 (79%)	59 (80%)	
Yes	15 (21%)	15 (20%)	
**Chest pain**			0.049
No	50 (68%)	61 (82%)	
Yes	23 (32%)	13 (18%)	
**Chest distress**			0.953
No	48 (66%)	49 (66%)	
Yes	25 (34%)	25 (34%)	
**Symptoms**			0.736
No	56 (77%)	55 (74%)	
Yes	17 (23%)	19 (26%)	

^1^n (%), ^2^Pearson’s Chi-squared test.

### Performance of the models

**Fig. 3:**
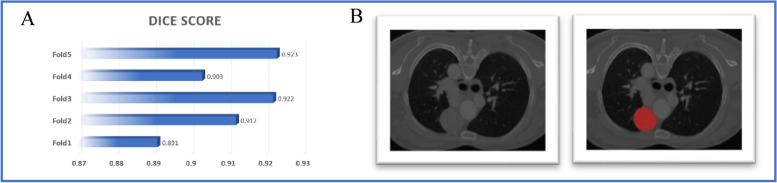
The performance of segmentation and the result of CT image segmentation. **A** The dice scores for each fold in the five-fold cross-validation. **B** The left image shows the CT scan before segmentation, while the right image shows the result after segmentation

For segmentation network, the average validation dice coefficients for each fold were 0.891, 0.912, 0.922, 0.903, and 0.923, respectively, with an overall average value of 0.910 (Fig.3A). The trained nnU-Net segmentation network was used to segment ROIs from the CT images. The segmentation result was shown in Fig.3B. The nnU-Net parameters were as follows: 'epoch': '1000', 'batch_size': 2, 'data_identifier': 'nnU-NetPlans_3d_fullres', 'patch_size': [48, 224, 224], 'spacing': [1.0, 1.0, 1.0], 'normalization_schemes': ['CTNormalization'], 'UNet_class_name': 'PlainConvUNet', 'n_conv_per_stage_encoder': [2, 2, 2, 2, 2, 2], 'n_conv_per_stage_decoder': [2,2, 2, 2, 2], 'num_pool_per_axis': [3, 5, 5], 'conv_kernel_sizes': [[3, 3, 3], [3, 3, 3], [3, 3, 3], [3, 3, 3], [3, 3, 3], [3, 3, 3]].

**Fig. 4:**
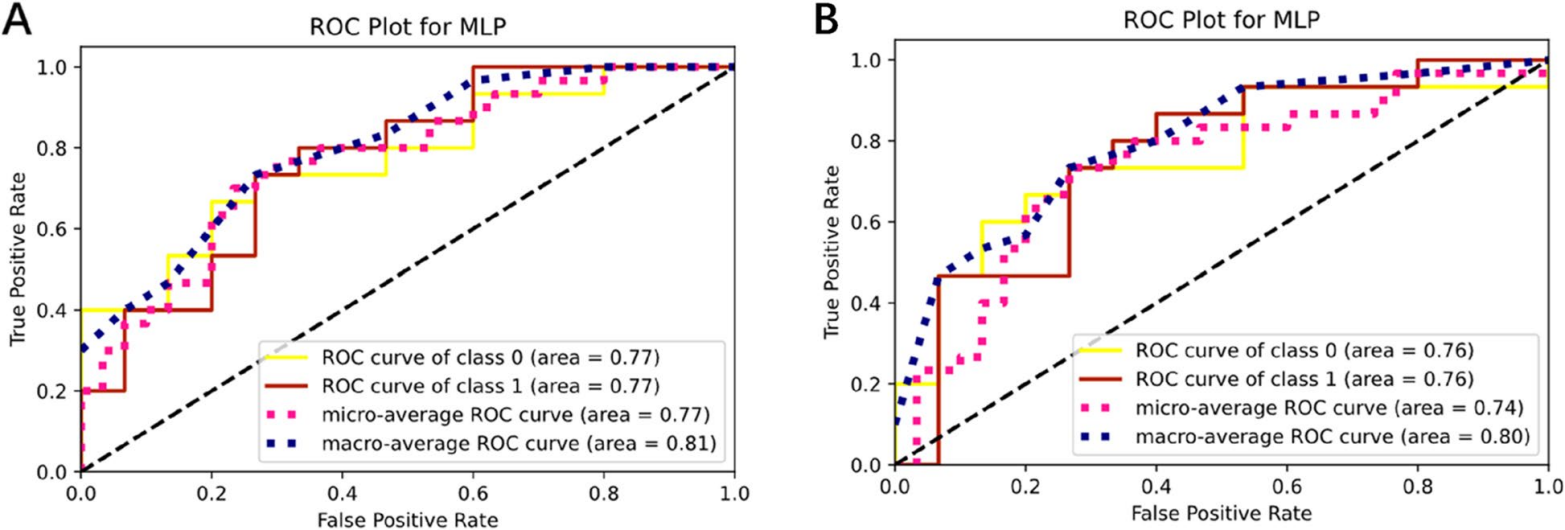
Validation and evaluation of radiomic model and deep model (2D). **A** ROC curve and AUC of radiomic model. **B** ROC curve and AUC of deep model (2D)

**Fig. 5:**
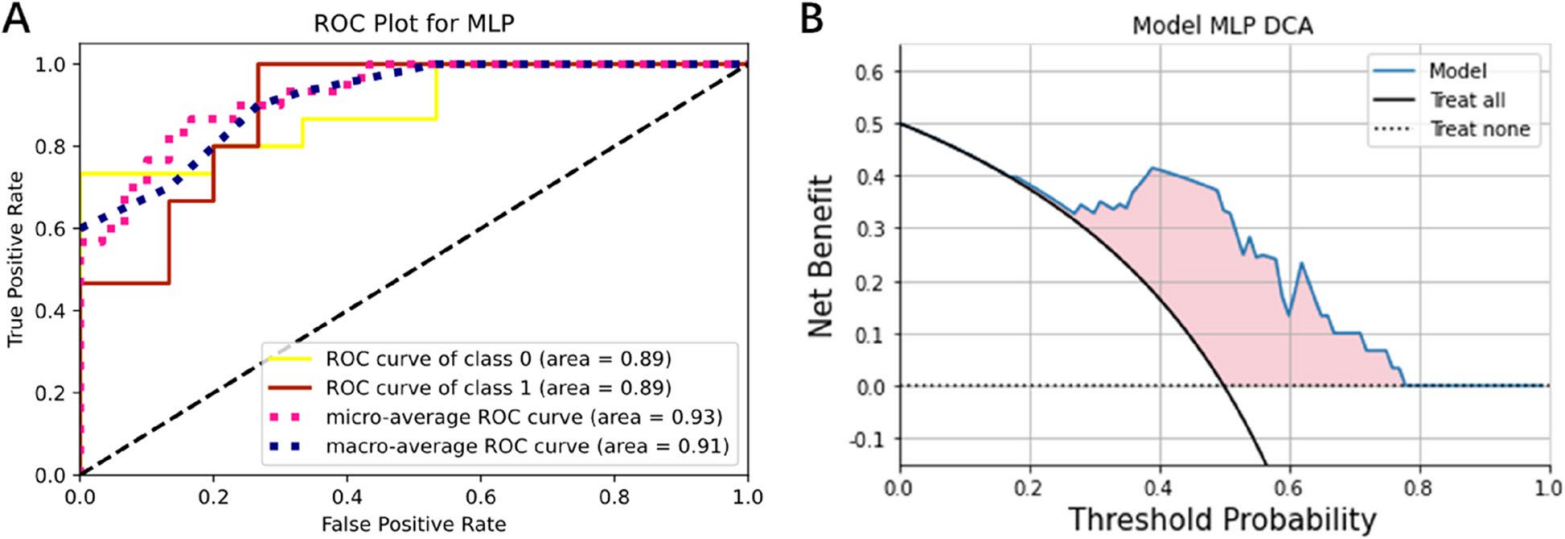
Validation and evaluation of deep model (**A**) ROC curves and AUC (**B**) DCA of deep model

**Fig. 6:**
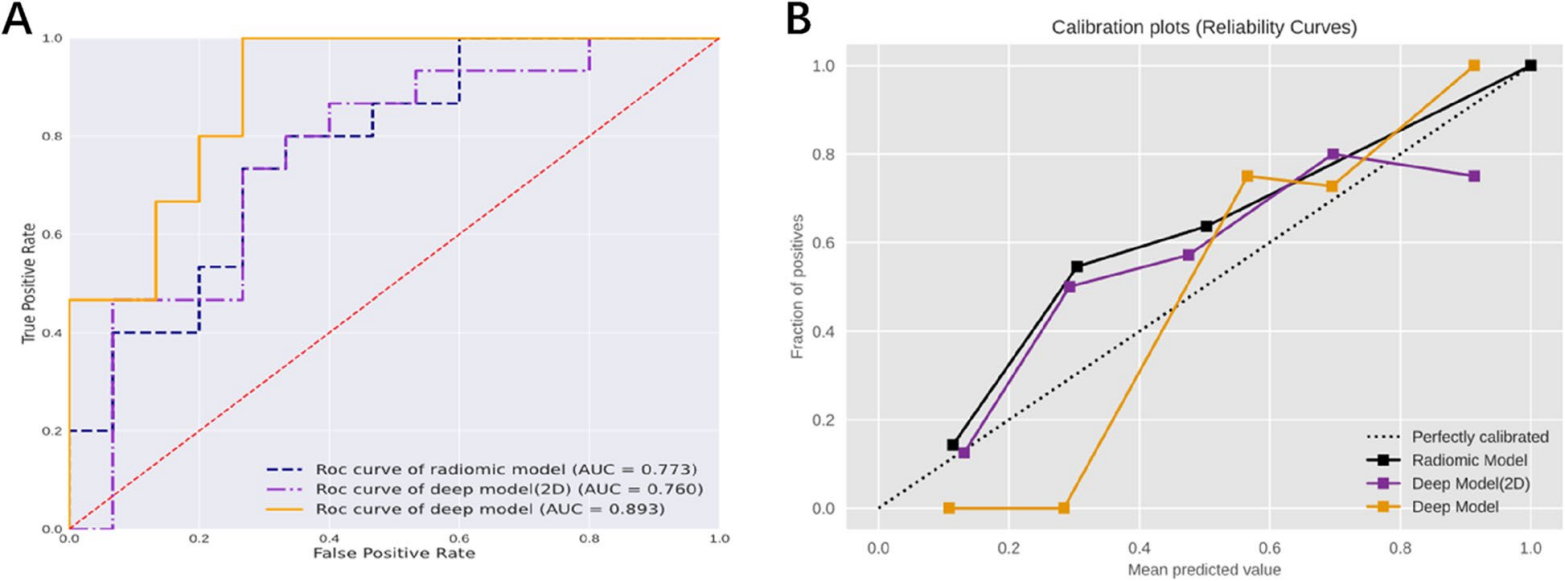
Performance comparison of radiomic model, deep model (2D) and deep model. **A** Comparison of ROC curves for three models. **B** Comparison of calibration curve for three models

For prediction network, the deep model achieved an AUC of 0.998 (95% CI 0.995-1.000) in the training cohort, along with an accuracy of 0.974, a sensitivity of 1.00, and a specificity of 0.966. In comparison, the radiomics model obtained an AUC of 0.773 (95% CI 0.688–0.858) and an accuracy of 0.692, the deep model (2D) obtained an AUC of 0.981(95% CI 0.964–0.998) and an accuracy of 0.897. In the testing cohort, the radiomics model’s AUC was 0.769 (95%CI: 0.599–0.938) (Fig. 4A), and the AUC of deep model (2D) was 0.760 (95%CI: 0.579–0.942) (Fig. 4B). The deep model achieved a better AUC of 0.893 (95% CI: 0.778-1.000), with accuracy, sensitivity, and specificity of 0.833, 1.000, and 0.733, respectively. The ROC curve was shown in Fig. 5A, and the DCA showed a better net benefit than radiomic model (Fig. 5B). A comparison of the performance of three models was shown in Fig. 6A and B, and detailed data about AUC, accuracy, sensitivity, and specificity of the models were presented in Table 2.

**Table 2:** The prediction performance of three models

Model	AUC (95% CI)	Accuracy	Sensitivity	Specificity	Task
Radiomic Model	0.773(0.688–0.858)	0.692	0.655	0.814	Train
	0.769(0.599–0.938)	0.700	0.800	0.600	Test
Deep Model (2D)	0.981(0.964–0.998)	0.897	0.966	0.881	Train
	0.760(0.579–0.942)	0.733	0.800	0. 714	Test
Deep Model	0.998(0.995- 1.000)	0.974	1.000	0.966	Train
	0.893(0.778- 1.000)	0.833	1.000	0.733	Test

*AUC *Area under the receiver operating characteristic curve,* CI *Confidence interval

## Discussion

At present, contrast-enhanced CT stands as the preferred preoperative examination for evaluating thymomas. Our study shows the potentials of utilizing preoperative CT images to distinguish subtype high risk and low risk of thymoma, which might potentially in assisting surgical procedures and clinical decision making. About the prediction of thymoma risk, previous studies have used radiomic features of CT images to differentiate between high and low risk. In their study, Dong et al. [30] utilized contrast-enhanced computed tomography (CE-CT) images to establish a radiomic model and a combined model for predicting the risk categorization of thymomas. They achieved AUCs of 0.819 and 0.870, respectively. Ozkan et al. [31] proposed a machine-learning model and assessed its ability to classify low and high risk thymomas on a small CT dataset. They achieved an AUC of 0.83 using the MLP Classifier. On the other hand, Yang et al. [32] attempted to develop a classification model for thymoma risk using deep learning. They utilized a 3D-DenseNet model based on deep learning (DL) to distinguish between stage I and stage II thymomas and achieved an AUC of 0.773.

In our study, the framework was capable of delineating the tumor ROI and extracting 3d deep learning features. This method significantly reduces the time and workload of medical professionals, and automated processes could provide consistent results, reducing the risk of human error or variance between different healthcare professionals. Additionally, this study has demonstrated the efficacy of utilizing 3D deep features extracted from CT images, affirming its capacity to enhance predictive performance. The deep learning model, which utilizes 3D deep features and clinical characteristics, achieved a higher AUC of 0.893 in the independent testing cohort. This was superior to the radiomic model, which was based on radiomic features and clinical characteristics, that achieved an AUC of 0.769. Similarly, the model using 2D deep features and clinical characteristics yielded an AUC of 0.760. The deep model has exhibited enhanced performance. When juxtaposed with the baseline radiomic model and deep model (2D), this deep model elevated the AUC by 16.1pt and 17.5pt on the testing set respectively. The results demonstrated that the deep model exhibited excellent prediction capabilities and deep features could capture subtle changes and characteristics in the images, including information that radiomic features and 2D features couldn’t capture. Furthermore, clinical features such as gender and the presence of chest pain were found to be potentially relevant to the study outcomes. However, further analysis was required to determine their significance in relation to the research objectives.

After analyzing the calibration curve, it became evident that the deep model exhibited superior reliability and validity. The decision curve analysis (DCA) illustrated that the deep model for thymoma risk stratification yielded more advantages compared to either the treat-all or treat-none strategy at various threshold probabilities. Furthermore, it showed better clinical utility. The comprehensive analysis of all results underscored the potential benefits of the deep model in stratifying thymoma patients, particularly in clinical settings where it aids clinicians in making accurate diagnoses.

However, during data collection, we made every effort to include as many thymoma patients as possible, while also trying to cover all subtypes of thymoma. Despite these efforts, our dataset may still have certain limitations in terms of representativeness. Thus, validation within a multi-center study, employing a larger sample size, becomes imperative for this framework. In addition, elevating the transparency and interpretability of the comprehensive deep model could further enhance its practical utility.

## Conclusions

This study proposed a deep model for risk stratification of thymoma pathological subtypes, which integrated clinical characteristics and 3D deep features extracted from arterial phase CT images. The model demonstrated consistent predictive abilities and achieved higher performance compared to the radiomic model and deep model (2D). The findings from this study were anticipated to make a significant contribution towards enhancing the early detection and prediction of thymoma. Moreover, they were expected to offer clinicians a more precise risk assessment, facilitating informed treatment decision-making and improving patients’ prognostic evaluation.

### Supplementary Information

Supplementary Material 1.

## Data Availability

The dataset from Shengjing hospital was used under approval for the current study. Restrictions apply to the availability of this dataset and so it is not publicly available.

## Abbreviations

LASSO: Least absolute shrinkage and selection operator
AUC: Area under curve
CI: Confidence intervals
DCA: Decision curve analysis
ROC: Receiver operating characteristic curve

## References

[1] https://www.cancer.org/cancer/types/thymus-cancer/about/key-statistics.html.

[2] WHO Classification of Tumours Editorial Board. Thoracic Tumours: WHO Classification of Tumours 5th. WHO Press. 2021.

[3] Jeong YJ (2004). Does CT of thymic epithelial tumors enable us to differentiate histologic subtypes and predict prognosis?. Am J Roentgenol.

[4] Altabella L (2022). Machine learning for multi-parametric breast MRI: radiomics-based approaches for lesion classification. Phys Med Biol.

[5] Poirion OB (2021). DeepProg: an ensemble of deep-learning and machine-learning models for prognosis prediction using multi-omics data. Genome Med.

[6] Jiang B (2022). Deep learning reconstruction shows better lung nodule detection for ultra-low-dose chest CT. Radiology.

[7] Cheng B (2022). Predicting EGFR mutation status in lung adenocarcinoma presenting as ground-glass opacity: utilizing radiomics model in clinical translation. Eur Radiol.

[8] Heydarheydari S, Birgani MJT, Rezaeijo SM (2023). Auto-segmentation of head and neck tumors in positron emission tomography images using non-local means and morphological frameworks. Pol J Radiol.

[9] Peng Z (2021). Application of radiomics and machine learning in head and neck cancers. Int J Biol Sci.

[10] Zhang K, Liu X, Shen J, et al. Clinically Applicable AI System for Accurate Diagnosis, Quantitative Measurements, and Prognosis of COVID-19 Pneumonia Using Computed Tomography. Cell. 2020;181(6):1423–33. 10.1016/j.cell.2020.04.045 PMC7196900 32416069

[11] Rezaeijo SM, Chegeni N, Baghaei Naeini F, Makris D, Bakas S (2023). Within-modality synthesis and novel radiomic evaluation of brain MRI scans. Cancers (Basel).

[12] Khanfari H, Mehranfar S, Cheki M, Mohammadi Sadr M, Moniri S, Heydarheydari S, Rezaeijo SM (2023). Exploring the efficacy of multi-flavored feature extraction with radiomics and deep features for prostate cancer grading on mpMRI. BMC Med Imaging.

[13] Kayicangir A (2021). CT imaging-based machine learning model: a potential modality for predicting low-risk and high-risk groups of thymoma: “Impact of surgical modality choice”. World Journal of Surgical Oncology.

[14] Wang D (2022). Histological classification and invasion prediction of thymoma by machine learning-based computed tomography imaging. Contrast Media Mol Imaging.

[15] Liu J, et al. CT-Based Radiomics Signatures for Predicting the Risk Categorization of Thymic Epithelial Tumors. Front Oncol. 2021. 10.3389/fonc.2021.628534. 10.3389/fonc.2021.628534 PMC7953900 33718203

[16] Shang. et al, Machine-learning classifiers based on non-enhanced computed tomography radiomics to differentiate anterior mediastinal cysts from thymomas and low-risk from high-risk thymomas: A multicenter study. Front Oncol. 2022. 10.3389/fonc.2022.1043163. 10.3389/fonc.2022.1043163 PMC9731806 36505817

[17] Nakajo M (2022). The efficacy of ^18^F-FDG-PET-based radiomic and deep-learning features using a machine-learning approach to predict the pathological risk subtypes of thymic epithelial tumors. Br J Radiol.

[18] Fedorov A (2012). 3D slicer as an image computing platform for the quantitative imaging network. Magn Reson Imaging.

[19] Isensee F, Jaeger PF, Kohl SAA, Petersen J, Maier-Hein KH. nnU-Net: a self-configuring method for deep learning-based biomedical image segmentation. Nat Methods. 2021;18(2):203–11. 10.1038/s41592-020-01008-z. 10.1038/s41592-020-01008-z 33288961

[20] Boehm KM (2022). Multimodal data integration using machine learning improves risk stratification of high-grade serous ovarian cancer. Nat Cancer.

[21] Wang G (2021). A deep-learning pipeline for the diagnosis and discrimination of viral, non-viral and COVID-19 pneumonia from chest X-ray images. Nat Biomed Eng.

[22] K He, et al. Deep Residual Learning for Image Recognition. IEEE Conference on Computer Vision and Pattern Recognition(CVPR). 2016.

[23] Sihong Chen, et al. Med3D: Transfer Learning for 3D Medical Image Analysis.arXiv preprint arXiv:1904.00625. 2019. 10.48550/arXiv.1904.00625.

[24] Windeatt T (2006). Accuracy/diversity and ensemble MLP classifier design. IEEE Trans Neural Netw.

[25] Wang T (2022). Radiomics for survival risk stratification of clinical and pathologic stage IA pure-solid non-small cell lung cancer. Radiology.

[26] Moons KG, Altman DG, Reitsma JB (2015). Transparent reporting of a multivariable prediction model for individual prognosis or diagnosis (TRIPOD): explanation and elaboration. Ann Intern Med.

[27] Kim H (2020). CT-based deep learning model to differentiate invasive pulmonary adenocarcinomas appearing as subsolid nodules among surgical candidates: comparison of the diagnostic performance with a size-based logistic model and radiologists. Eur Radiol.

[28] Xueyi Z, et al. Deep learning radiomics can predict axillary lymph node status in early-stage breast cancer. Nature Communications. 10.1038/s41467-020-15027-z. 10.1038/s41467-020-15027-z PMC7060275 32144248

[29] Gao W (2022). A predictive model integrating deep and radiomics features based on gadobenate dimeglumine-enhanced MRI for postoperative early recurrence of hepatocellular carcinoma. Radiol Med.

[30] Dong W, et al, Application of a combined radiomics nomogram based on CE-CT in the preoperative prediction of thymomas risk categorization. Front Oncol. 2022. 10.3389/fonc.2022.944005. 10.3389/fonc.2022.944005 PMC9446086 36081562

[31] Ozkan, et al. Combined clinical and specific positron emission tomography/computed tomography-based radiomic features and machine-learning model in prediction of thymoma risk groups. Nucl Med Commun. 2022;43(5):529–539. 10.1097/MNM.0000000000001547. PMID: 35234213. 10.1097/MNM.0000000000001547 35234213

[32] Yang L (2020). Development of a deep learning model for classifying thymoma as Masaoka-Koga stage I or II via preoperative CT images. Ann Transl Med.

