# Supplementary material for: Deep learning for risk stratification of thymoma pathological subtypes based on preoperative CT images
Source: BMC Cancer. 2024 May 28;24:651. doi: 10.1186/s12885-024-12394-4 (PMC11134708; doi:10.1186/s12885-024-12394-4)
Supplement: Supplementary file 1 — Supplementary Material 1. [file 12885_2024_12394_MOESM1_ESM.docx]

**Supplementary Table 1**

Model parameters and hyperparameters

| Model | Paramete and hyperparameter |
| --- | --- |
| PCA | n_components:128  svd_solver: auto |
| LASSO | lambda=0.045 |
| nnU-Net | Pixel size of the input images: [127, 512, 512]  epoch: 1000  batch_size: 2  patch_size: [48, 224, 224]  spacing: [1.0, 1.0, 1.0]  conv_kernel_sizes: [3, 3, 3] |
| Resnet 50(3D) | Pixel size in the 3DCNN: [3,224, 224, 224]  CT: window width of 350 and a window level of 40  Voxel size of 1*1*1 mm,  Input dimensions are 224x224x224 |
| Resnet 50 (2D) | Pixel size:[3,224,224] |
| MLP | hidden_layer_sizes= (128, 64, 32)  max_iter=300  solver='sgd'  random_state=0 |
